# Artificial intelligence in South African universities: curriculum transformation and decolonisation—aid or obstacle?

**DOI:** 10.3389/fsoc.2025.1543471

**Published:** 2025-07-08

**Authors:** Charles Maimela, Palesa Mbonde

**Affiliations:** Faculty of Law, University of Pretoria, Pretoria, South Africa

**Keywords:** artificial intelligence, higher education, curriculum transformation, decolonization, 4th industrial revolution, inclusive education, South African universities, #FeesMustFall

## Abstract

The integration of Artificial Intelligence (AI) in South African universities presents both opportunities and challenges, particularly within the context of curriculum transformation and decolonisation. This paper critically examines the relevance of AI in relation to the #FeesMustFall movement, which advocates for equitable access to education, and explores how these themes intersect with decolonisation efforts in South Africa. Although AI technologies promise advantages like tailored learning experiences, improved administrative processes, and enhanced research capabilities, they also present issues related to epistemic bias, digital disparities, and the reinforcement of Western-centric knowledge systems. Grounded in empirical research, this study investigates whether AI serves as an aid or an obstacle in South African higher education, with a specific focus on Historically White Universities (HWUs) and Historically Black Universities (HBUs). Using the Diffusion of Innovation (DOI) theory as a framework, the research explores disparities in AI adoption across institutions, analysing infrastructural constraints, policy gaps, and the broader implications of AI for knowledge production. The findings reveal that while HWUs have made significant strides in AI integration due to better funding and international collaborations, HBUs continue to face systemic barriers that hinder equitable access to AI-driven learning tools. Moreover, AI’s reliance on Western datasets and epistemologies risks perpetuating digital colonialism, complicating ongoing efforts to decolonise the curriculum. This paper underlines the urgent need for Afrocentric AI models that align with local contexts and values, inclusive policy frameworks that address the needs highlighted by #FeesMustFall, and targeted investments in digital infrastructure. By doing so, it aims to ensure that AI contributes meaningfully to higher education curriculum transformation and decolonisation in South Africa.

## 1 Introduction

The integration of Artificial Intelligence (AI) in higher education is a growing phenomenon worldwide, with universities leveraging AI-driven technologies for teaching, learning, research, and administration (Luckin et al., 2016). In South Africa, this shift coincides with broader curriculum transformation and decolonisation debates, which seek to address historical inequalities in higher education and redefine knowledge production (Le Grange, 2016). While AI has the potential to enhance learning through personalised education, intelligent tutoring systems, and predictive analytics, concerns persist regarding its Western-centric biases, epistemic injustices, and accessibility disparities. This paper critically examines the role of AI in South African universities within the framework of curriculum transformation and decolonisation, questioning whether AI serves as an aid or an obstacle in these processes.

### 1.1 Background and context

#### 1.1.1 South African universities landscape

South Africa’s higher education landscape has undergone significant transformation, evolving from a racially segregated system under apartheid to a more inclusive and diversified structure post-1994. Historically, higher education institutions were designed to serve the interests of the white minority, leading to the establishment of universities that reinforced racial disparities reflecting the deeply entrenched segregation of the time (Bunting, 2006).

In the democratic era, the South African government initiated a comprehensive restructuring of the higher education system to promote equity, efficiency, and responsiveness to societal needs. This led to the consolidation of institutions through mergers and incorporations, resulting in a more streamlined system comprising 26 public universities (Cloete and Moja, 2005) (According to Essop, 2020). These institutions are categorised into five distinct types:

1. Research-Intensive Universities (RIUs): These universities focus heavily on research and postgraduate education. They include institutions like the University of Cape Town and University of Pretoria (UP).
2. Other Universities (OUs): This category encompasses institutions that offer a broad range of undergraduate and postgraduate programmes but may not have the same research intensity as the RIUs. They include institutions like North-West University (NWU) and University of Johannesburg (UJ).
3. Historically Black Universities (HBUs): Established primarily to serve black students during apartheid, these universities have played a crucial role in providing access to higher education for historically marginalised communities. The HBUs include but are not limited to the University of Fort Hare (UFH), University of Limpopo (UL).
4. Universities of Technology (UoTs): Focused on vocational and technical education, UoTs offer programmes that are career-oriented and aligned with industry needs. The UoTs include institutions like Cape Peninsula University of Technology (CPUT), Tshwane University of Technology (TUT), and Vaal University of Technology (VUT).
5. Distance University: The University of South Africa (Unisa) is the sole dedicated distance education institution in the country, providing flexible learning opportunities to a diverse student body.

This categorisation reflects the government’s efforts to create a diversified higher education system that caters to various educational needs and promotes equitable access. In South Africa there is also a distinction between private and public institutions of higher education. According to CHE (Council on Higher Education) (2022). By 2019, South Africa’s higher education sector comprises 26 public universities, 131 private institutions, 50 public TVET colleges, 287 private colleges, and 9 CET colleges, reflecting its expansion and diversification.

#### 1.1.2 Historically black vs. historically white universities

Historically Black Universities (HBUs) and Historically White Universities (HWUs) in South Africa remain distinct due to their historical origins, funding disparities, and institutional capacities. HBUs were established under apartheid to serve Black students, often with limited resources, inadequate infrastructure, and restricted academic offerings (Badat, 2010). These institutions were designed to reinforce racial segregation and limit Black access to higher education, ensuring their marginal participation in the economy (Bunting, 2006). In contrast, HWUs were well-funded, equipped with superior facilities, and positioned as centres of academic excellence, producing the country’s elite workforce (Jansen, 2004).

Despite democratic reforms, inequalities persist. HWUs continue to benefit from historical advantages, including better funding, stronger research output, and international collaborations, making them more competitive in global rankings. Meanwhile, HBUs struggle with financial instability, lower student retention rates, and challenges in attracting qualified academic staff due to budgetary constraints [CHE (Council on Higher Education), 2016]. The student demographics further reflect these disparities; while HWUs have diversified, they still cater largely to privileged students with greater access to financial resources and academic support (Wolpe, 1995).

HBUs, on the other hand, remain critical in serving students from disadvantaged backgrounds, but many grapple with overcrowding, outdated curricula, and insufficient government support (DHET, 2020). While policies like the National Student Financial Aid Scheme (NSFAS) aim to level the playing field, systemic inequalities continue to hinder HBUs from achieving parity with HWUs. Without targeted structural interventions, these historical disparities will likely persist.

#### 1.1.3 Impact of #FeesMustFall and COVID-19 on higher education

The #FeesMustFall movement, which erupted in 2015, initially focused on the demand for free higher education but also ignited a broader conversation on decolonizing the curriculum in South African universities. Student activists argued that the inherited colonial and Eurocentric curriculum failed to reflect African epistemologies, histories, and realities (Heleta, 2016). This push for decolonization led to institutional debates about the inclusion of indigenous knowledge systems, African scholarship, and the restructuring of academic programmes to reflect local contexts (Heleta, 2016). While some universities responded by revising course content and introducing African-centred scholarship, systemic changes remain slow due to entrenched institutional cultures and academic resistance (Mbembe, 2016).

The COVID-19 pandemic further disrupted higher education, forcing universities to shift to emergency remote teaching (ERT) to sustain academic activities. Universities like UNISA used methods such as Iris invigilation software programme and the invigilator app which students would need to set up either on a computer or smartphone to continue the academic programme during the pandemic (Baboolal-Frank, 2022).

The rapid digital transformation exposed deep inequalities, as many students, particularly those from historically disadvantaged backgrounds, lacked access to devices, stable internet, and conducive learning environments (Czerniewicz et al., 2020). Universities adopted various interventions, such as zero-rated educational websites and laptop distribution, yet the digital divide persisted, disproportionately affecting students at HBUs (Mohohlwane et al., 2021). Despite these challenges, the pandemic accelerated the adoption of digital learning tools and blended learning approaches, reshaping higher education’s future. While ERT was a temporary solution, it laid the foundation for long-term digital transformation, emphasising the need for accessible, inclusive, and resilient education systems in South Africa.

#### 1.1.4 Debates on transformation vs. decolonisation in South Africa

The debates on transformation and decolonisation in South African higher education remain central to addressing historical injustices and shaping inclusive academic spaces. While the concepts are interconnected, they diverge in scope and ideological grounding. Transformation broadly refers to structural, institutional, and policy-driven changes aimed at redressing inequalities and creating more inclusive institutions (Badat, 2010). It encompasses increasing diversity in student and staff demographics, revising curricula to reflect South Africa’s socio-political realities, and ensuring equitable resource distribution (Jansen, 2017). Decolonisation, on the other hand, extends beyond policy reform to a radical epistemic shift that challenges Eurocentric knowledge systems and advocates for African-centred scholarship (Mbembe, 2016).

Where transformation focuses on integrating historically excluded groups into existing institutional structures, decolonisation questions the very foundations of these structures, arguing that they perpetuate colonial power dynamics (Le Grange, 2016). Critics argue that transformation alone risks assimilation without fundamentally altering the epistemic hierarchy that privileges Western knowledge (Heleta, 2016). The 2015–2016 #FeesMustFall and #RhodesMustFall movements exemplified this tension, with students demanding both institutional transformation and the decolonisation of knowledge production (Ndlovu-Gatsheni, 2018). While transformation is often state-driven, decolonisation remains a grassroots intellectual and activist struggle, advocating for dismantling colonial legacies rather than merely reforming them.

#### 1.1.5 Overview of curriculum transformation in South Africa

Curriculum transformation involves intentional and systemic changes to educational curricula (Shay, 2015). In South Africa, curriculum transformation is a critical and ongoing process aimed at addressing historical inequalities and aligning education with the country’s socio-political and economic needs. The apartheid-era education system was characterised by racial segregation and inequality, with curricula designed to reinforce social stratification (Jansen, 1998). In response, post-1994 education reforms sought to dismantle these legacies through policies promoting inclusivity, critical engagement, and relevance to the broader African context [DHET (Department of Higher Education and Training), 2013].

At the higher education level, curriculum transformation has been driven by the need to decolonize knowledge production, diversify faculty representation, and integrate African epistemologies (Le Grange, 2016). The #FeesMustFall and #RhodesMustFall movements intensified debates on curriculum reform, emphasising the need for the incorporation of indigenous knowledge systems (Heleta, 2016). While universities have made strides in revising curricula, disparities remain in implementation, particularly between historically white and black institutions. The transformation process continues to evolve, requiring sustained collective determination, institutional commitment, and stakeholder collaboration to achieve meaningful and lasting change.

#### 1.1.6 Artificial intelligence in South African universities

The integration of Artificial Intelligence (AI) into South African universities has been a progressive journey, reflecting a broader commitment to technological advancement in higher education. However, determining the exact time of AI’s initial adoption in South Africa is challenging due to sparse documentation and its gradual integration into academic and research settings. This integration is influenced by both global technological trends and the country’s unique socio-political environment. Early AI initiatives in South African higher education trace back to the late 1990s and early 2000s, with pioneering research and development emerging from institutions such as the University of Pretoria and the University of the Witwatersrand (University of Witwatersrand, 2024). These universities focused on areas like machine learning, natural language processing, and robotics, laying the groundwork for AI’s role in academia. Over the years, AI adoption has transcended technical faculties, permeating various aspects of university operations. AI-driven tools are now employed to enhance personalised learning experiences, automate administrative tasks, and analyse student performance data. For instance, the University of Pretoria’s Computational Intelligence Research Group (CIRG) established in 2003 & based in the Department of Computer Science focuses on research in the broad realm of computational intelligence. The research group has been instrumental in developing AI applications that support both academic and administrative functions (Ferrein and Meyer, 2012). The COVID-19 pandemic further accelerated AI integration, as institutions sought innovative solutions to the challenges of remote learning. AI-powered platforms have been used to maintain student engagement and provide virtual support, ensuring continuity in education during unprecedented times (Funda and Mbangeleli, 2024).

Despite these advancements, the implementation of AI in South African universities is not without challenges. Issues such as digital inequality, data privacy concerns, and ethical considerations persist. The disparity in technological infrastructure between well-resourced and historically disadvantaged institutions aggravates existing inequalities, potentially limiting the benefits of AI to a portion of the student population.

Moreover, the ethical implications of AI, including potential biases in algorithms and the handling of sensitive data, necessitate the development of comprehensive policies (Chen, 2023). Current higher education policies in South Africa often lack explicit guidelines addressing these concerns, emphasising the need for a robust policy framework that ensures responsible AI usage.

While AI holds significant promise for transforming South African higher education by enhancing learning outcomes and operational efficiency, its integration must be approached thoughtfully. Addressing infrastructural disparities, ethical issues, and policy gaps is crucial to harnessing AI’s potential as a tool for inclusive and equitable education.

#### 1.1.7 Problem statement

The integration of Artificial Intelligence (AI) in South African universities presents both opportunities and challenges for curriculum transformation and decolonisation. While AI can enhance teaching, learning, and research, its adoption is uneven, with Historically White Universities (HWUs) benefiting from better infrastructure and funding, while Historically Black Universities (HBUs) face financial and technological barriers. This disparity reinforces existing inequalities and deepens epistemic divides (Cloete, 2018; Marwala, 2021).

AI technologies are largely developed within Western paradigms, often excluding African epistemologies and indigenous knowledge systems (Heleta, 2016). This raises concerns about cultural bias and digital colonialism, as AI may reinforce Eurocentric curriculum instead of supporting decolonisation (Oluwaseyi and Victoria, 2024). Additionally, movements like #FeesMustFall and disruptions such as COVID-19 have highlighted systemic inequities, emphasising the need for inclusive AI policies (Booysen, 2016; Czerniewicz et al., 2020).

This paper examines whether AI aids or hinders curriculum transformation and decolonisation in South African universities. By comparing AI adoption across institutions, it assesses AI’s potential as a tool for equitable, context-sensitive reform or as a force that exacerbates historical divides.

#### 1.1.8 Research aim & objectives

The aim for this paper is to evaluate AI’s role as a facilitator or a hindrance to curriculum transformation and decolonisation efforts in South African universities, examining disparities in adoption between Historically White and Historically Black Universities.

##### 1.1.8.1 Objectives

- To analyse the current landscape of South African universities, categorising institutions based on historical and structural differences in AI adoption.
- To examine the relationship between AI, curriculum transformation, and decolonisation, assessing whether AI-driven innovations align with or contradict transformation goals.
- To compare AI adoption and integration across HWUs and HBUs, identifying key disparities in infrastructure, funding, and digital capacity.

#### 1.1.9 Significance of the study

This research is vital to understanding AI’s impact on higher education transformation in South Africa. It highlights digital divides, contributes to the discourse on epistemic justice, and offers policy recommendations to ensure AI aligns with African knowledge systems. As South Africa navigates the Fourth Industrial Revolution, this study informs stakeholders on leveraging AI for inclusive and decolonial curriculum reform.

## 2 Theoretical perspective: diffusion of innovation (DOI) theory

The Diffusion of Innovation (DOI) Theory, developed by Everett Rogers (1962, 2003), explains how new ideas, technologies, and practices spread within a social system. The theory posits that innovation adoption occurs through a process influenced by individual, organisational, and societal factors. This framework is widely used to analyse technology adoption in education, including the integration of artificial intelligence (AI) in university curricula (Rogers, 2003; Sahin, 2006).

According to Rogers (2003), the diffusion process follows five key stages:

1. Knowledge—Exposure to innovation and its functionalities.
2. Persuasion—Formation of a positive or negative attitude towards innovation.
3. Decision—Commitment to adopting or rejecting the innovation.
4. Implementation—Application of the innovation in real contexts.
5. Confirmation—Reinforcement or discontinuation of the innovation based on outcomes.

These stages are influenced by perceived attributes of the innovation, such as relative advantage, compatibility, complexity, trialability, and observability, which shape adoption patterns in higher education institutions (HEIs) (Straub, 2009).

### 2.1 Application of DOI theory to AI adoption in South African universities

Innovation Attributes and AI Adoption in Higher Education.

The adoption of AI in South African universities can be analysed through DOI’s five perceived attributes:

- Relative Advantage—AI enhances personalised learning, automates administrative processes, and improves research capabilities (Marwala, 2021). However, its benefits are not uniformly accessible due to disparities between Historically White Universities (HWUs) and Historically Black Universities (HBUs) (Mtshweni, 2022).
- Compatibility—AI’s compatibility with existing curricula, teaching practices, and institutional policies influences its uptake. While HWUs have integrated AI-driven research tools, HBUs face challenges related to infrastructure, faculty training, and curriculum alignment (Czerniewicz et al., 2020).
- Complexity—AI’s technical complexity hinders adoption, particularly in underfunded institutions where faculty lack specialised expertise in machine learning, data science, and AI ethics.
- Trialability—The ability to experiment with AI-driven tools affects adoption rates. Universities with dedicated AI research centres, such as Wits who have the “The Wits MIND Institute.” have better access to pilot programmes and industry partnerships, accelerating adoption (University of Witwatersrand, 2024).
- Observability—The visible impact of AI in improving learning outcomes, administrative efficiency, and research innovation encourages wider adoption. However, institutions without adequate digital infrastructure struggle to observe tangible benefits.

### 2.2 Categories of AI adopters in universities

Rogers (2003) categorises adopters into five groups, which can be mapped onto AI adoption in South African higher education institutions:

- Innovators (2.5%)—Leading AI research institutions, such as Wits University and UCT, which actively engage in AI-driven learning and research collaborations.
- Early Adopters (13.5%)—Universities with moderate AI integration, such as Stellenbosch and Pretoria, leveraging AI for student engagement and research.
- Early Majority (34%)—Universities experimenting with AI but facing financial and training barriers, including some comprehensive universities.
- Late Majority (34%)—Institutions with minimal AI integration due to resource limitations, such as some HBUs.
- Laggards (16%)—Universities that resist AI adoption due to financial, infrastructural, and pedagogical challenges.

### 2.3 Institutional barriers and DOI’S organisational context

DOI theory also considers organisational structures as key determinants of innovation diffusion (Greenhalgh et al., 2004). In South African universities, barriers to AI adoption include:

- Financial constraints—HBUs have lower research funding, limiting AI integration.
- Policy and governance—AI policies are inconsistent, leading to unequal AI adoption rates.
- Resistance to change—Institutions unfamiliar with AI may resist its adoption due to technological scepticism.
- Digital divide—Unequal computational resources and internet access hinder AI implementation in previous disadvantaged institutions.

### 2.4 DOI theory and the future of AI in higher education

DOI theory provides a useful lens for understanding the uneven adoption of AI in South African universities. While HWUs act as early adopters, HBUs face systemic barriers that slow diffusion. For AI to contribute meaningfully to curriculum transformation and decolonization, universities must address policy gaps, digital inequalities, and faculty training needs. Inclusive AI strategies that align with South Africa’s higher education transformation goals must be promoted to champion AI as an aid rather than a hindrance.

## 3 Literature review

This literature review aims to provide a comprehensive overview of the existing research on the role of Artificial Intelligence (AI) in higher education, curriculum transformation and or decolonisation, and the socio-political context of South African universities. By critically evaluating previous studies, the review identifies gaps in the literature, highlights emerging debates, and establishes the relevance of this study within a rapidly evolving academic field.

The review discusses key themes, including broader South Africa and AI, South African Universities and AI: challenges and opportunities it presents within the South African context, historical perspectives on curriculum transformation decolonisation, and recent investigations into the intersection of AI and curriculum transformation. This sets the foundation for understanding AI’s role in shaping educational equity and decolonisation.

### 3.1 Broader South Africa and artificial intelligence

Artificial Intelligence (AI) is increasingly influencing various sectors in South Africa, offering both opportunities and challenges. In the manufacturing industry, AI adoption has led to significant improvements in productivity, quality control, and supply chain management. A study by Nzama et al. (2024) highlights that AI technologies facilitate advanced quality control processes, error detection, and prevention, resulting in reduced waste and enhanced efficiency. The integration of AI into supply chain operations has optimised inventory management and demand forecasting, contributing to overall operational excellence.

In the public sector, AI presents opportunities to enhance human resource management (HRM) by automating routine tasks, thereby allowing HR professionals to focus on strategic decision-making. Chilunjika et al. (2022) discuss how AI can minimise biases in recruitment and selection processes, promoting a more equitable workforce. However, the potential displacement of jobs due to AI automation raises concerns about employment and necessitates proactive policy measures to reskill and upskill the existing workforce.

The healthcare sector in South Africa stands to benefit from AI through improved diagnostics, patient care, and operational efficiency. Naidoo et al. (2022) propose that, despite the promise of AI in healthcare, existing policy frameworks are inadequate to foster innovation in this field. They recommend developing a national policy framework that addresses issues such as outdated legislation, data and algorithmic bias, workforce impact, liability concerns, and the need for innovation in AI systems tailored to the South African context.

From a broader perspective, Brokensha et al. (2023) argue for a humanistic approach to AI in Africa, emphasising the need for solutions that consider the continent’s unique socio-economic realities. They advocate for decolonial strategies that move beyond Eurocentric models, addressing disparities related to gender, race, labour, and power. This approach aims to ensure that AI development is inclusive and beneficial to all segments of African society.

while AI offers transformative potential across various sectors in South Africa, realising its benefits, the South African government prioritised the development of a guiding framework. The Department of Communications and Digital Technologies (DCDT) has been at the forefront of AI regulation in South Africa. Following the publication of its National AI Plan in April 2024, the DCDT has taken a further leap by releasing the South African National AI Policy Framework. The National AI Policy will be the foundation for creating AI regulations and potentially an AI Act in South Africa. The policy aligns with global AI governance standards to achieve these goals and addresses the nation’s socioeconomic disparities. The implementation and specific regulations governing AI in higher education remain in its infancy.

### 3.2 South African universities and AI: challenges and opportunities

The integration of AI into South African higher education presents both opportunities and challenges. AI-driven solutions can enhance access to education, particularly for under-resourced institutions, by providing adaptive learning tools (Corrigan et al., 2023). However, unless tailored to South Africa’s socio-economic disparities, AI risks deepening inequalities rather than addressing them. The “digital divide” remains a critical concern, as AI could democratise learning but also alienate disadvantaged groups lacking digital literacy or infrastructure. Ensuring equitable access to digital tools is crucial for AI’s effective implementation.

South Africa’s higher education landscape, shaped by colonial and apartheid histories, remains marked by disparities in funding and resources (Akinwalere and Ivanov, 2022). Historically White Universities (HWUs) are better positioned to integrate AI due to superior infrastructure and funding (Patel and Ragolane, 2024; Mhlanga and Moloi, 2020), whereas Historically Black Universities (HBUs) struggle with outdated equipment and insufficient support. This geographic divide influences students’ learning experiences and future opportunities, reinforcing structural inequalities (Akinwalere and Ivanov, 2022). Research highlights the risk of AI implementation further entrenching a Eurocentric curriculum if not carefully contextualised (Eubanks, 2018; Noble, 2018).

AI offers significant potential for expanding educational access. AI-driven platforms can facilitate distance learning and adaptive education, particularly in rural areas with limited infrastructure (Arinto, 2016; Meet, 2024). However, without integration into decolonial frameworks, AI may reinforce existing inequities (Akinwalere and Ivanov, 2022). Ethical concerns also arise, as biased algorithms could marginalise underrepresented groups and perpetuate exclusionary practices (Noble, 2018).

To harness AI’s benefits while mitigating its risks, institutions must address geographic disparities, digital literacy gaps, and curriculum biases. A deliberate, inclusive approach can ensure AI serves as a tool for transformation rather than perpetuating historical inequalities.

### 3.3 Curriculum transformation in South African universities

The intersection of AI and curriculum transformation in South Africa is an emerging field of inquiry, with scholars beginning to explore how AI can both aid and hinder decolonisation efforts. Akinwalere and Ivanov (2022) contends that AI has the potential to facilitate curriculum transformation by providing adaptive learning environments that cater to diverse student needs. AI-driven systems, when designed inclusively, can support more flexible, personalised approaches to learning, which may align with decolonial objectives.

Zembylas (2021), argues that AI systems in education must be designed with ethical considerations at their core, particularly when dealing with decolonisation. The tendency of AI to replicate biases of the data it is trained on, often drawn from Global Northern sources, poses significant challenges for universities in the Global South, which aim to develop curricula that are inclusive of diverse knowledge systems.

Research by Akinwalere and Ivanov (2022) explores the integration of AI in higher education, emphasising both its potential benefits and challenges. AI can enhance educational outcomes by personalising learning experiences and streamlining administrative functions, promoting efficiency and inclusivity. However, they also caution that AI poses risks such as data privacy concerns, ethical issues, and the potential for reinforcing inequalities if not properly contextualised. In the context of curriculum transformation, AI’s role in personalising learning channels based on student data is significant, but for it to genuinely support decolonisation, it must prioritise African knowledge systems rather than merely adapting Western models. This nuanced approach ensures that AI fosters rather than impedes educational equity and decolonial efforts in South Africa (Akinwalere and Ivanov, 2022).

However, as Ally and Perris (2022) and Benjamin (2019) highlight, AI technologies are not neutral. Without a critical, context-sensitive approach to their implementation, these tools may unintentionally reinforce existing hierarchies of knowledge. Scholars argue that the introduction of AI in South African universities must be closely aligned with local needs and decolonial priorities to avoid perpetuating the very inequalities that curriculum transformation seeks to address (Noble, 2018).

Emerging research suggests that AI could also play a role in improving access to decolonised curricula by enabling the development of digital repositories that house African knowledge systems and materials (Patel and Ragolane, 2024). This could ensure that a wider audience has access to indigenous knowledge and provide a platform for the dissemination of decolonised content across institutions. However, further research is needed to explore how AI can be leveraged to support the co-creation of knowledge that transcends traditional Eurocentric academic frameworks.

This review highlights the complexity of integrating AI into curriculum transformation and decolonisation efforts within South African universities. While AI offers promising opportunities for inclusive education and fostering curriculum transformation, it also presents significant risks, particularly if it is implemented without attention to historical inequalities and local contexts. The review highlights the necessity of a critical, nuanced approach to AI adoption that prioritises educational equity and aligns with the broader goals of decolonisation. Future research should focus on the dynamics of AI implementation in diverse institutional contexts, exploring strategies to ensure that technological advancements complement and support decolonial educational reform.

## 4 Methodology

### 4.1 Research design

This study employs a qualitative research approach, using document analysis to examine AI-related institutional documents from UP and UFH. Document analysis enables a systematic review of AI related institutional materials to identify themes and trends in AI adoption within higher education.

Qualitative research is particularly suited for understanding the meanings and experiences, enabling a nuanced examination of the disparities in resource availability, infrastructure, and progress made in curriculum transformation and decolonisation (Yin, 2008). The study’s research design is a qualitative case study, which allows for an in-depth exploration of the complex dynamics and contextual factors influencing the integration of AI at these institutions (Merriam, 1998). A comparative case study design is adopted to understand how these two universities, each with distinct historical and educational contexts are integrating AI into their curricula. This approach facilitates an exploration of institutional differences concerning policy adoption, infrastructure, and AI-driven pedagogical strategies.

### 4.2 Data collection

The study relies on primary sources, specifically official university documents related to AI integration. The following documents were analysed:

#### 4.2.1 University of Pretoria

1. Student’s Guide: Leveraging Generative Artificial Intelligence for Learning: This guide educates students on effectively using generative AI tools to enhance their learning experiences while emphasising ethical considerations and academic integrity (University of Pretoria, 2023).
2. Lecturer’s Guide: Leveraging Generative Artificial Intelligence for Teaching and Learning Enhancement: Aimed at lecturers, this document explores the potential of generative AI to improve teaching methodologies and learning outcomes, providing practical insights into AI integration in pedagogy (University of Pretoria, 2024).
3. Guide for ChatGPT Usage in Teaching and Learning: This guide discusses the impact of AI tools like ChatGPT on higher education, offering guidelines for their responsible use in teaching and learning contexts (University of Pretoria, 2024).
4. Ethical Conduct Including Plagiarism and Artificial Intelligence: UP’s updated policy underscores the institution’s commitment to academic integrity, addressing the ethical use of AI tools and the importance of avoiding plagiarism (University of Pretoria, n.d).

#### 4.2.2 University of Fort Hare

1. Research Ethics Policy: This policy outlines ethical standards for research activities at UFH, which may encompass considerations relevant to AI research and applications (University of Fort Hare, 2025).

### 4.3 Data analysis

A thematic analysis is employed to identify patterns in how AI is being implemented in curriculum transformation at UP and UFH. The Diffusion of Innovation (DOI) Theory (Rogers, 2003) serves as the theoretical framework to analyse how AI adoption differs across institutional contexts. Key themes to be explored include:

- Institutional Readiness for AI Integration: Examining funding, digital infrastructure, and staff expertise.
- AI and Curriculum Transformation: Evaluating how AI is shaping course content, teaching methodologies, and student engagement.
- AI and Decolonization: Investigating whether AI contributes to or hinders decolonial curriculum efforts.
- Barriers to AI Adoption: Identifying challenges such as financial limitations, epistemic bias, and access disparities.

The comparative study aims to highlight institutional disparities in AI adoption, considering factors such as funding, strategic investments, and infrastructural constraints.

### 4.4 Ethical considerations

As the study is based on document analysis, there are no ethical concerns related to human participants. However, care will be taken to ensure the credibility and authenticity of the sources by relying on official university documents and policies. The research will adhere to ethical guidelines for secondary data analysis, ensuring that all sources are properly cited and interpreted objectively.

### 4.5 Limitations

The primary limitation of this study is that document analysis does not capture lived experiences or stakeholder perspectives on AI adoption. Future research could incorporate interviews or surveys with faculty and students to gain firsthand insights into AI’s impact on curriculum transformation and decolonization. However, given the focus on institutional policies and structural analysis, document analysis remains an appropriate method for this study.

## 5 Discussion and findings

### 5.1 The unequal adoption of AI in South African universities

A key finding of this study is that AI adoption in South African universities remains highly uneven, reflecting historical inequalities between Historically White Universities (HWUs) and Historically Black Universities (HBUs). HWUs such as UP have made significant strides in AI research and integration into their curricula, with dedicated AI research centres, industry partnerships, and well-funded computer science faculties. In contrast, HBUs such as the University of Fort Hare face major challenges, including limited funding, infrastructure constraints, and lack of AI expertise among faculty (Mtshweni, 2022).

The disparities in AI adoption are rooted in the historical funding structures of higher education in South Africa, where HWUs received greater state investment during the apartheid era and have continued to attract private sector and international research grants (Cloete, 2018). This is evident in the fact that AI-driven learning tools, machine learning programmes, and digital research platforms are more prevalent in well-resourced universities, while HBUs struggle with even basic digital access for students (DHET, 2022).

To illustrate, The University of Pretoria (UP) and the University of Fort Hare (UFH) have both incorporated Artificial Intelligence (AI) and digital tools into their educational frameworks, though their approaches differ in scope and implementation. UP has actively embraced AI through initiatives such as The SCU-B ‘Scooby, ‘an AI-powered chatbot designed to support student wellbeing (University of Pretoria) UP employs Blackboard “A web-based course management system that allows educators and learners to participate in the online delivery of classes or material” (Maimela, 2024) called (clickUP) as its Learning Management System (LMS), integrating AI-driven tools like Blackboard Ally for accessibility, Blackboard Assist for centralised resources, and Gradescope for AI-assisted grading. Additionally, UP employs Pyramid Analytics to aggregate student performance data and facilitate early interventions.

In contrast, UFH also employs Blackboard as its LMS, offering lecture notes, recorded lessons, and engagement forums. While specific AI policies and grading tools at UFH are not explicitly detailed, the institution emphasises technology-enhanced learning and has established assessment and plagiarism policies (University of Fort Hare) Unlike UP, there is no available information on UFH’s implementation of AI-powered student support tools or data analytics for student monitoring. Overall, UP has taken a more structured approach to AI integration, while UFH focuses on broader digital learning strategies without explicitly defined AI-driven initiatives. Recently, the University of Fort Hare hosted a conference on Artificial Intelligence (AI) from September 10th to 12th, 2024, highlighting AI’s potential to transform teaching, learning, and research (University of Fort Hare, 2024). While this event marked a significant step in the university’s engagement with AI, it also emphasised the lack of tools, resources, and established guidelines available to HBUs like UFH when compared to better-resourced institutions such as UP.

### 5.2 Funding and infrastructure challenges

Historically White Universities (HWUs) benefit from private and international funding, allowing them to expand AI research and integrate advanced technologies into their curricula (Oluwaseyi and Victoria, 2024). In contrast, Historically Black Universities (HBUs) face significant financial constraints that hinder their ability to adopt AI-driven learning tools and research initiatives, further exacerbating institutional disparities (Marwala, 2021). These funding imbalances contribute to stark differences in student access to AI-enabled learning platforms, reflecting broader digital divides in South African higher education. While students at well-resourced institutions can engage with AI-powered educational tools, those at underfunded universities struggle with limited digital infrastructure, reinforcing existing inequalities in learning opportunities and academic success.

### 5.3 AI and the transformation-decolonisation debate

The study also finds that AI complicates ongoing debates about transformation and decolonisation in South African higher education. Transformation aims at making curricula more inclusive and representative, while decolonisation seeks to replace Eurocentric knowledge systems with African-centred epistemologies (Heleta, 2016; Luckett and Shay, 2020). AI, being predominantly developed in Western contexts, reinforces global knowledge hierarchies and may contradict efforts to decolonize curricula.

AI-driven educational tools and platforms are predominantly trained on Western datasets, which marginalises African epistemologies and limits the representation of indigenous knowledge systems (Faloye and Ajayi, 2021). This epistemic imbalance is not new; as Chisholm (2005, p. 194) asserts, “through surfacing this knowledge, hidden and suppressed reservoirs of cultural knowledge come into being that challenge the Eurocentric and rationalist assumptions of school-based knowledge.” In this light, the integration of AI must be carefully scrutinised for its potential either to reinforce or disrupt these entrenched knowledge hierarchies.

This bias is further exacerbated by Natural Language Processing (NLP) models, which often struggle with African languages, thereby reinforcing the dominance of English in higher education and restricting access for non-English speakers (Marivate, 2020). Additionally, most AI ethics frameworks applied in South Africa are developed in the Global North, failing to account for the country’s unique socio-political contexts and perpetuating external epistemic influences that may not align with local educational and cultural needs (Mudaly, 2018).

Some scholars argue that AI could be repurposed for decolonial goals, for example, by developing AI models trained on African linguistic and cultural data. However, such efforts require substantial investment and policy interventions, which are currently lacking (Marwala, 2021).

### 5.4 AI and the legacy of #FeesMustFall

The #FeesMustFall movement was a defining moment in the transformation of South African higher education, demanding free, decolonised education and addressing economic barriers to access (Booysen, 2016). This research finds that AI does not inherently resolve these issues and, in some cases, exacerbates educational inequalities.

### 5.5 AI as a barrier to access

Students from underprivileged backgrounds face significant challenges in accessing AI-driven learning tools due to financial and infrastructural barriers (Czerniewicz et al., 2020). The shift to online AI-enhanced learning during the COVID-19 pandemic further exacerbated these disparities, disproportionately disadvantaging students at Historically Black Universities (HBUs), where limited digital resources and inadequate connectivity hindered effective engagement with remote learning platforms (Mhlanga and Moloi, 2020). Digital inequality remains a persistent issue across South African higher education institutions, as access to AI-based learning tools is unevenly distributed, with well-resourced universities benefiting from advanced technologies while underfunded institutions struggle to keep pace (DHET, 2021).

While AI offers potential benefits such as personalised learning and adaptive assessments, it risks perpetuating socio-economic inequalities if access is not democratised (Mudaly, 2018). Without targeted interventions, AI will primarily benefit students at well-resourced universities, leaving marginalised groups further behind.

### 5.6 The need for Afrocentric AI in higher education

To address the challenges identified in this study, there is a pressing need for Afrocentric AI models and frameworks. These models should Incorporate African languages and indigenous knowledge systems. Be developed with ethical frameworks that reflect African socio-cultural values and prioritise equitable access through government-funded AI infrastructure in HBUs.

## 6 Recommendations

Based on the findings of this study, several recommendations are proposed to enhance the integration of Artificial Intelligence (AI) in South African universities while ensuring alignment with curriculum transformation and decolonisation objectives.

### 6.1 Developing Afrocentric AI models and inclusive digital infrastructure

Most AI tools used in South African universities are developed outside the continent, leading to algorithmic biases and a lack of contextual relevance (Faloye and Ajayi, 2021). To address this, South African universities should prioritise the development of locally tailored AI models that integrate African languages, histories, and knowledge systems to ensure that AI-driven education aligns with the country’s diverse epistemological landscape. Establishing partnerships with African AI research institutions is crucial for creating context-specific datasets that accurately reflect South African knowledge systems and cultural nuances. Additionally, AI-driven educational tools must be designed to support multilingual learning, with a particular emphasis on incorporating indigenous languages to promote inclusivity and accessibility in higher education. Government bodies such as the Department of Higher Education and Training (DHET) and the Council on Higher Education (CHE) should provide funding incentives to support these initiatives, ensuring historically disadvantaged institutions (HDIs) receive priority investment (Department of Higher Education and Training, 2015).

### 6.2 Bridging the digital divide between historically black and historically white universities

The study highlights inequities in AI adoption, with historically white universities (HWUs) leading AI integration due to stronger financial and research capacity, while historically black universities (HBUs) lag behind due to resource constraints (Cloete, 2018). To bridge this gap, the DHET and private sector partners should provide targeted funding and infrastructure support to HBUs to enhance their AI research capabilities (Mtshweni, 2022). There should be the establishment of inter-university AI hubs that allow resource-sharing between HBUs and HWUs, ensuring equitable AI research and development opportunities and finally universities should implement public-private partnerships to ensure low-income students have access to AI-driven learning tools and digital platforms (Mhlanga and Moloi, 2020).

### 6.3 Embedding AI in curriculum transformation frameworks

The integration of AI into curriculum transformation efforts should align with broader decolonisation goals, ensuring that AI-driven education does not reinforce Eurocentric knowledge hierarchies (Heleta, 2016). Universities should revise curriculum policies to ensure AI adoption aligns with South African cultural and socio-political contexts (Le Grange, 2016). They should also incorporate AI ethics courses that critically examine issues such as data colonialism, bias in machine learning, and the global AI knowledge economy (Corrigan et al., 2023) and Promote indigenous knowledge systems in AI research and development to foster epistemic justice in higher education (Mudaly, 2018).

### 6.4 Policy interventions for ethical AI development and governance

AI in higher education must be ethically governed to prevent exacerbating inequalities in access, data privacy, and academic freedom (Marwala, 2021). To ensure responsible AI implementation, policymakers should develop national AI policy frameworks that outline ethical guidelines for AI use in universities, addressing issues such as algorithmic bias, data privacy, and student autonomy. Establish AI regulatory bodies within higher education institutions to monitor and evaluate AI adoption in curricula, assessment methods, and research practices and encourage open-access AI research initiatives, ensuring that AI tools developed within South Africa remain accessible to all universities rather than concentrated in elite institutions.

### 6.5 Strengthening AI literacy and academic capacity building

A key challenge in AI adoption is the lack of AI literacy and specialised expertise among faculty and students. Universities should provide AI training programmes for lecturers to ensure faculty can effectively integrate AI tools into their teaching methodologies. Establish AI literacy modules as part of general education requirements, ensuring students gain foundational knowledge in AI and digital skills (DHET, 2022). Offer funding for postgraduate AI research, particularly for students from historically disadvantaged backgrounds, to enhance diverse participation in AI scholarship.

## 7 Conclusion

The integration of Artificial Intelligence (AI) in South African universities presents a double-edged sword in the broader discourse on curriculum transformation and decolonisation. While AI has the potential to enhance personalised learning, improve accessibility, and streamline administrative processes, its Western-centric development models and the digital divide between historically black universities (HBUs) and historically white universities (HWUs) present challenges to equitable implementation (Faloye and Ajayi, 2021).

The historical inequalities in South African higher education, exacerbated by apartheid-era funding disparities, have had a lasting impact on university infrastructure and research capacity HWUs, such as UP have led AI research and adoption, benefiting from international partnerships and private sector funding (Mtshweni, 2022). In contrast, many HBUs, like the University of Fort Hare, continue to struggle with financial constraints, inadequate digital infrastructure, and lower research output, hindering their ability to integrate AI into curricula effectively.

The FeesMustFall movement and the COVID-19 pandemic have highlighted the structural inequalities in the education system, highlighting the urgent need for policy interventions that ensure AI-driven education benefits all students, regardless of institutional background (Booysen, 2016; Czerniewicz et al., 2020). Furthermore, the debate between transformation and decolonisation complicates AI adoption, as many AI tools reinforce Eurocentric knowledge hierarchies rather than supporting African epistemologies (Heleta, 2016). To maximise AI’s role as an aid rather than an obstacle, South African universities must adopt contextualised AI policies that prioritise:

- Investing in digital infrastructure at HBUs to bridge the technological gap.
- Developing Afrocentric AI models that incorporate indigenous knowledge systems.
- Enhancing AI literacy among faculty and students to ensure meaningful engagement with the technology.
- Strengthening collaborations between government, universities, and private sector stakeholders to support equitable AI adoption.

Ultimately, AI can either reinforce existing inequalities or serve as a catalyst for curriculum transformation—the outcome depends on how institutions navigate the intersection of technology, historical inequalities, and epistemic justice (Marwala, 2021). Addressing these challenges requires intentional policy frameworks that align AI with South Africa’s broader higher education transformation agenda while ensuring that technological advancements do not perpetuate past injustices (DHET, 2021).

## Data Availability

The original contributions presented in the study are included in the article/supplementary material, further inquiries can be directed to the corresponding author/s.
